# Cognitive and emotional effects of renal transplantation

**DOI:** 10.4103/0019-5545.31614

**Published:** 2006

**Authors:** A.A. Pawar, J. Rathod, S. Chaudhury, S.K. Saxena, D. Saldanha, V.S.S.R. Ryali, K. Srivastava

**Affiliations:** * Senior Adviser (Psychiatry); ** Clinical Psychologist; **** Professor and Head, RINPAS; *** Resident, INHS Asvini, Mumbai; ***** Professor and Head; ****** Associate Professor; ******* Scientist E, Department of Psychiatry, Armed Forces Medical College, Pune

**Keywords:** Renal transplantation, cognitive function, depression, life satisfaction

## Abstract

**Background:**

Recent studies have shown a high prevalence of depression and cognitive changes in patients with end-stage renal disease (ERSD) and renal transplant recipients. There are few data available on the cognitive and emotional changes in patients undergoing renal transplantation in India.

**Aim:**

To evaluate the changes in cognitive profile and depression in renal transplant recipients.

**Methods:**

Thirty consecutive patients undergoing renal transplantation were evaluated 1 month before and 3 months after successful renal transplant with Beck Depression Inventory (BDI), Weschler Adult Performance Intelligence Scale (WAPIS), Luria Nebraska Neuropsychological battery (LNNB) and Life satisfaction scale.

**Results:**

Our study revealed an 86.7% prevalence of depression in ESRD patients as compared to 56.7% in post renal transplant patients. Analysis of neurocognitive functions on LNNB did not reveal any significant impairment. Furthermore, analysis of the Life satisfaction scale revealed most of the patients scored high satisfaction levels despite the stress of their disease. Results on WAPIS brought out significant improvement in intelligence quotient (IQ) after renal transplantation.

**Conclusion:**

Successful renal transplant is associated with improvement in depression, IQ and life satisfaction.

## INTRODUCTION

The incidence of renal failure is 200 cases in a million people each year. Most of the illnesses causing kidney failure are not in older people, but usually in young adults. Dialysis and kidney transplantation are the two basic treatments for chronic renal failure. Peritoneal dialysis and haemodialysis are the two main forms of dialysis. In India, haemodialysis is the preferred option used by most centres. Haemodialysis imposes severe restrictions on the patient and his family. The patient is placed in a situation where he is totally dependent on a machine and medical personnel two or three times a week. He needs a strict diet and multiple daily medications. Water intake is reduced to sucking a piece of ice. The cost of the treatment is prohibitive and the loss of working days due to treatment adds to the financial stress. Depressed mood and depressive syndromes are common occurrences in these patients.^1^ This, is understandable since depression commonly follows loss, and these patients lose their independence, strength, job and energy. The evaluation of depression is complicated by the fact that the symptoms of end-stage renal disease (ESRD) such as diminished appetite, loss of energy, constipation and diminished sexual ability resemble the somatic symptoms of depression. Presence of anhedonia, poor self-esteem, crying spells, helplessness and suicidal ideation are better indicators of the disease. Organic brain syndromes such as delirium and dementia are also common in these patients. Patients involved in intellectual work notice progressive impairment in their intellectual abilities as the day of dialysis approaches.^2^,^3^ Renal transplantation is usually considered the treatment of choice. A successful transplant dramatically improves the quality of life of the patient. The patient is free from dependence on a machine, dietary restriction and loss of workdays due to dialysis. Cognitive function is said to improve and in children growth and development returns to normal levels.^4^

Transplantation has its own problems. The patients are on life-long immunosuppresants such as steroids, which have their own side-effects. The fear of rejection looms large and the patient still has to be under regular medical supervision. Some workers have not found any difference between transplanted patients and those on dialysis on measures of psychological adjustment and vocational rehabilitation.^5^ In view of the contradictory findings and paucity of Indian studies in this field, the present study was undertaken to find out the prevalence of depressive symptoms, cognitive function and Life satisfaction in haemodialysis patients and to assess the changes in these parameters following a successful renal transplant.

## METHODS

All patients with ESRD admitted to the renal centre of a tertiary care service hospital and being worked-up for renal transplant were included in the study. The patients (*n*=30) had been on regular haemodialysis for at least 6 months.

### Inclusion criteria

- Patients diagnosed as ESRD who were on regular dialysis and were awaiting surgery
- Age >18 years and <60 years

### Exclusion criteria

- Age <18 and >60 years
- Coexisting medical illnesses such as ischaemic heart disease diabetes (IHD), asthma, hepatitis, coexisting infections and head injuries
- Past or family history of psychiatric disorders

After selection and before administration of the tests, an informed consent was taken from the patients. The patients were evaluated 1 month before undergoing transplantation and 3 months after renal transplantation. Demographic details including age, sex, region, religion and diet were recorded on a specially designed proforma. The following tests were administered 1 month before and 3 months after transplantation:

- Beck Depression Inventory II (BDI)^6^
- Luria Nebraska Neuropsychological Battery (LNNB)^7^
- Weschler Adult Performance Intelligence Scale (WAPIS)^8^
- Life Satisfaction scale^9^

The tests were administered in more than one session to avoid exhaustion and boredom in the patients. The tests were scored as per the test booklets. The results were tabulated and analysed using SPSS software.

## RESULTS

The mean age of the ESRD patients was 39.6 years. There were 28 male and 2 female patients. The educational profile revealed that all were educated at least up to class X. Most of the patients undergoing renal transplant were Hindus. About 50% were vegetarians.

Table 1 shows that 86.7% of the pre-transplant cases were depressed (score >9 on BDI) and this fell to 56.7% after transplant. An important finding was that 60% of the pre-transplant cases were moderately to severely depressed while none of patients were moderately to severely depressed after renal transplant. The average BDI score was 22.03 in this population and the score decreased significantly to 9.83 following renal transplantation. We also subdivided the BDI score into cognitive affective components taking the first 13 statements, which is said to be a more accurate measure of depressive symptoms in physically ill patients. The average score on the cognitive scale was 15.01 and this reduced to 4.68 after transplant, while the somatic score reduced from 7.02 to 5.15 underlining the fact that the effect of renal transplant was mainly on the cognitive affective component of BDI.

**Table 1:** BDI scores of the patients before and after renal transplant (*n*=30)

BDI score	Pre-transplant	Post-transplant
Not depressed (0-9)	4 (13.3%)	13 (43.3%)
Mildly depressed (10-16)	8 (26.7%)	17 (56.7%)
Moderately depressed (17-29)	10 (33.3%)	0
Severely depressed (>29)	8 (26.7%)	0
Mean score	22.03	9.83**
Cognitive affective BDI	15.01	4.68
Somatic BDI	7.02	5.15

** p<0.01 highly significant (Wilcoxon sign rank test)

Table 2 shows that in ESRD patients cognitive affective symptoms such as feelings of punishment, crying, being self-critical, indecisiveness, past failure and worthlessness were common. Among the somatic symptoms, loss of energy was a common symptom. Suicidal ideation was not present in any patient. Post-transplant, the sense of failure showed a mild increase, but all the other symptoms either decreased or remained the same.

**Table 2:** Symptom profile on the BDI before and after renal transplant

Description	Pre-transplant *n* (%)	Post-transplant *n* (%)
Feelings of punishment	17 (56.7)	10 (33.3)
Self-critical	13 (43.3)	8 (26.7)
Crying spells	13 (43.3)	5 (16.7)
Worthlessness	12 (40.0)	2 (6.7)
Loss of energy	12 (40.0)	6 (20.0)
Indecisiveness	10 (33.3)	2 (6.7)
Sense of failure	10 (33.3)	11 (37.3)
Sadness	8 (26.7)	1 (3.3)
Pessimism	6 (26.7)	4 (13.3)
Sleep disturbance	8 (26.7)	4 (13.3)
Irritability	8 (26.7)	7 (23.3)
Fatiguability	8 (26.7)	8 (26.7)
Loss of pleasure	7 (23.3)	3 (10)
Loss of interest	6 (20.0)	5 (16.7)
Self-hate	5 (16.7)	5 (16.7)
Difficulty in concentration	5 (16.7)	3 (10.0)
Guilt feelings	3 (10.0)	1 (3.3)
Agitation	2 (6.7)	2 (6.7)
Loss of appetite	2 (6.7)	1 (3.3)
Loss of libido	1 (3.3)	1 (3.3)
Suicidal thoughts	0 (0.0)	0 (0.00)

On the WAPIS, there was a highly significant increase in mean IQ scores after renal transplant (Table 3). Before transplant, 22 patients were in the average range for intelligence and 8 showed borderline retardation. After transplant, 2 moved to the bright–normal range while all the borderline retardation patients moved to the average range, thus indicating that this disease affects the intelligence. A significant difference was also observed between pre- and post-renal transplant cases in all the subscales of the WAPIS (Table 4).

**Table 3:** WAPIS (Indian version) scores of patients before and after renal transplantation

WAPIS score	Pre-transplant	Post-transplant
Mean IQ	88.5	101*
Bright-normal (110-119)	0 (0%)	2 (6.7%)
Average (85-109)	22 (73.3%)	28 (93.3%)
Borderline retardation (70-84)	8 (26.7%)	0 (0%)

* p<0.05 significant (Wilcoxon sign rank test)

**Table 4:** Scores obtained on performance subtests of WAPIS before and after renal transplant

WAPIS subtests	Pre-transplant	Post-transplant
Picture completion	7.40	9.87*
Digit symbol	8.77	11.27*
Block design	7.37	9.43*
Picture arrangement	8.83	9.90*
Object assembly	9.20	10.47*

* p<0.05 significant (Wilcoxon sign rank test)

The BDI scores of the ESRD patients did not correlate with the performance IQ except in the picture completion test (Table 5). After renal transplant, again the BDI scores did not correlate with the performance IQ except the block design test. This indicates that the lower IQ seen in patients before renal transplant was not due to the effect of depression as measured by the BDI.

**Table 5:** Correlation of BDI and WAPIS scores in patients before andafter renal transplant

BDI score		WAPIS subtests					
	
		Picture completion	Digit symbol	Block design	Picture arrangement	Object assembly	IQ performance
Pre-renal transplant	Pearson correlation	-0.406	-0.202	-0.242	-0.061	0.191	-0.195
	significance (2-tailed)	0.026*	0.285	0.197	0.750	0.311	0.302
Post-renal transplant	Pearson correlation	-0.060	-0.247	-0.388	-0.005	0.036	-0.254
	significance (2-tailed)	0.753	0.189	0.034*	0.977	0.849	0.175

* Indicates significance at 0.05 level

On the LNNB, significant differences were seen in motor functions, intellectual functions, receptive speech, expressive speech, visual functions, reading and memory following renal transplant (Table 6). There was no correlation between BDI and IQ with LNNB clinical subscales in the patients before and after renal transplant (Table 7).

**Table 6:** LNNB clinical scale scores before and after renal transplant

Clinical scale	Mean score	
	
	Pre-transplant	Post-transplant
Motor functions	35.43	31.50**
Intellectual functions	38.53	34.23*
Expressive speech	40.10	33.77**
Receptive speech	40.90	35.87**
Visual	38.77	33.33*
Tactile	41.83	39.30
Reading	49.10	40.37*
Writing	42.33	40.93
Arithmetic	46.43	45.93
Memory	38.57	34.90*

* p<0.05 significant;

** p<0.01 highly significant

**Table 7:** Correlation of LNNB scores with BDI and IQ before and after renal transplant

LNNB clinical scale		Pre-transplant		Post-transplant	
			
		BDI score	IQ	BDI score	IQ
Motor	Pearson correlation	-0.103	-0.222	-0.264	-0.097
	significance (2-tailed)	0.587	0.238	0.159	0.612
Intellectual	Pearson correlation	0.236	-0.144	-0.325	0.088
	significance (2-tailed)	0.210	0.448	0.080	0.645
Expressive speech	Pearson correlation	0.153	-0.019	-0.160	0.355
	significance (2-tailed)	0.419	0.922	0.397	0.054
Receptive speech	Pearson correlation	0.343	-0.108	0.096	0.132
	significance (2-tailed)	0.064	0.571	0.615	0.487
Visual	Pearson correlation	0.230	-0.025	-0.486	0.391
	significance (2-tailed)	0.222	0.897	0.006	0.033
Tactile	Pearson correlation	0.082	-0.222	0.054	0.021
	significance (2-tailed)	0.665	0.239	0.778	0.914
Reading	Pearson correlation	-0.076	-0.267	-0.041	0.150
	significance (2-tailed)	0.690	0.154	0.832	0.429
Writing	Pearson correlation	-0.057	-0.198	-0.163	-0.024
	significance (2-tailed)	0.767	0.295	0.389	0.899
Arithmetic	Pearson correlation	-0.136	-0.028	-0.116	0.169
	significance (2-tailed)	0.472	0.882	0.542	0.372
Memory	Pearson correlation	0.236	0.061	-0.070	-0.117
	significance (2-tailed)	0.209	0.750	0.713	0.536

On the Life satisfaction scale, 15 patients scored in the high range and fifteen in the average range before renal transplant (Table 8). All patients in the average group shifted to the high satisfaction group post-transplant. There was also a significant rise in the mean life satisfaction score after renal transplantation.

**Table 8:** Life satisfaction scale scores in patients before and after renal transplant

Life satisfaction scale	Patients	
	
	Pre-renal transplant	Post-renal transplant
High score (>135)	15	30
Average score (85-135)	15	0
Mean score	133	153*

* p<0.05 significant

## DISCUSSION

ESRD is a psychologically debilitating disease with considerable emotional morbidity. Depression is the most common symptom in these cases and has been attributed to the loss of many factors—the loss of independence, loss of working ability, loss of healthy self-image and loss or diminution of sexual ability.^1^,^10^ In this study, depression was present in as many as 86.7% of the total population before transplant (Table 1), the average score being 22.03 and decreasing to 9.83 after transplantation. Taking a cut-off of 17 to obviate the effects of somatic symptoms due to the illness from impinging on the BDI score, 60% of the population scored above the cut-off, indicating moderate to severe depression on the BDI before renal transplant. Similarly, a study of 62 Korean patients^11^ on chronic haemodialysis reported a 56.5% prevalence of depression following a cut-off of above 21. The score of 21 had been validated for the Korean population. We were unable to find any study validating the cut-off for the Indian population. They also validated the BDI score by interviewing the patients and applying the DSM-IV criteria. In their study, 34 out of 40 patients scoring more than 18 on the BDI met the criteria for major depressive disorder as per the DSM-IV. However, in our study none of the patients after renal transplant had a BDI score >17. This is unusual, as others have reported contrary findings.^2^ Teran–Escandon *et al*.^12^ reported a 40% prevalence of depression in their sample of post-transplant cases observed after 1 year. They also observed that patients who had received a kidney from live related donors had a lesser prevalence of depression than those who had received a kidney from cadavers. They hypothesized that patients with kidneys from live related donors might be overestimating their prognosis. In our sample, all the patients had received kidney from a live related donor, as cadaver transplants are not yet in vogue in India. Also, the patients in this study were evaluated 3 months after transplant, thus excluding cases of graft rejection. This may have led to a significant improvement in the symptoms of depression in the study.

The BDI proved to be a sensitive indicator for revealing depression and detecting the symptoms in a majority of cases. Out of the total population of 30 cases only 2 cases had been suspected of having depression by the treating nephrologist and sent for psychiatric evaluation. The reason for this could be that patients are reluctant to reveal psychological symptoms to the treating doctor unless these are specifically asked for. Depression has been thought to influence the nutritional status as indicated by albumin levels. Thus, poor nutritional status may mediate the relation between depression and mortality in ESRD.^13^ Depression in our study was not linked to the diet of the patient but was significantly linked to the educational level. Patients educated up to standard X showed more depression than those who had had more education. The large percentage of depression in post-renal transplant cases, who were apparently on the road to recovery, is a surprising finding. We attempted to analyse the reason for this by studying the subscales of the BDI. An analysis of the BDI subscales (Table 2) reveals that punishment feelings, guilty feelings, loss of energy, worthlessness and indecisiveness were the most common symptoms in ESRD patients. This substantiates the commonly held cultural belief that disease is a response to or punishment for wrongs committed earlier. Except for the loss of energy, which may be a symptom of the disease, other symptoms are psychological thus substantiating the validity of the BDI in these cases. Sexual difficulties have been reported to be common in these cases; however, in our study only 3.7% of the cases reported sexual difficulty. This may be due to the reluctance to accept and disclose sexual problems in our setting.

The common symptoms in the post-renal transplant group as reflected in the BDI were past failure, punishment feelings, crying and being self-critical. These symptoms also subs-tantially validate the effectiveness of the BDI as a tool for assessing depression in these cases. The high prevalence of depression in the pre- as well as the post-renal transplant cases may indicate the need for starting a prophylactic antidepressant in these cases. Trials of fluoxetine done earlier in such cases have shown effectiveness and considerable attenuation of symptoms by 8 weeks.^4^ Kramar *et al.*^14^ also found a 44% prevalence of depression in patients with ESRD, which is in agreement with our findings. Their study also linked depression to increased mortality which we were not able to verify. Depression is also reported to be higher in men^5^ and linked to depression in the spouse. We were unable to verify this in our study.

Of the patients 26.7% had a borderline IQ before renal transplant, while after transplant none of the patients had a borderline IQ. To negate the influence of culture and education we measured only the performance IQ using WAPIS. In a study of adults with ESRD since childhood^15^ the performance IQ was found to be 9.2 points lower than a comparative group of adults without renal disease. The lowest scores were observed in tasks that required concentration, memory and general knowledge. On analysing the subscales of WAPIS, in this study we found that the lowest scores were obtained in picture completion and block design. Picture completion is a measure of visual concentration and a non-verbal test of general information. A low score on this test may indicate difficulty in concentration and inadequate visual organization. Block design, on the other hand, is said to be a relatively non-verbal culture-free test of intelligence, which correlates highly with general intelligence. It is also said to be an excellent indicator of brain damage, especially of the right hemisphere.^16^ Low scores were obtained on other subtests as well which improved significantly after transplantation. In a similar study of 9 medically stable children and adolescents, improvement in intellectual functions was found after successful renal transplant.^17^ In another study involving 20 children and adolescents with ESRD before and after transplantation, patients exhibited significantly greater improvement from initial testing to 1 month after transplantation on the performance IQ and full-scale IQ.^18^ The significant difference was not maintained, however, at 1 year after transplantation. The later the onset of renal failure or the fewer the years in ESRD, the less the impairment in cognitive performance. Blood urea nitrogen, serum creatinine levels and blood pressure did not correlate with any of the cognitive or academic achievement measures. In our study too we did not find any such correlation. Ryan *et al.*^19^ compared patients on dialysis with a group of medical–psychiatric patients. They reported that renal cases showed greater deficit on object assembly and the block design subtest relative to neurological cases or medical—psychiatric patients. Thus, it appears that block design is a sensitive indicator of dysfunction in renal disease.

Analysis of the clinical scales of LNNB revealed significant differences between the patients before and after renal transplantation (Tables 6 and 7) in motor functions, intellectual ability, expressive and receptive speech, reading, memory and visual functions. It appears that language functions are impaired in patients with ESRD as there is impairment in both receptive and expressive speech. We were unable to come across any study in the literature where the LNNB had been applied. The Halstead Reitan Battery was administered by Souheaver *et al.*^20^ to 24 patients with advanced renal failure, 24 patients with neurological disorders and 24 patients with medical and/or non-psychotic psychiatric conditions. Their results indicated that the uraemic and neurological group was equal in the overall level of neuropsychological impairment and that both were significantly more impaired than the medical–psychiatric group. However, the uraemic group showed a pattern of deficits that was qualitatively different from both the neurological and medical–psychiatric groups. In a further study the group also compared patients on chronic haemodialysis with undialysed patients with uraemia and a third group of medical–psychiatric patients. The three groups of 16 patients each did not differ significantly in age, education, verbal intelligence, or degree of affective disturbance. Halstead–Reitan Battery subtest comparisons demonstrated that dialysis patients performed significantly better than uraemic patients and were equivalent to medical–psychiatric subjects on tasks of psychomotor problem-solving and spatial ability. Dialysis patients were impaired relative to the medical–psychiatric patients on a task of flexible thinking. Dialysis patients were impaired relative to medical–psychiatric subjects and equivalent to uraemic patients on tasks which required complex analysis, auditory information processing, language capacities, and sensory–perceptual functions. This impairment of language functions observed by the authors seems to be part of the uraemic process, which does not improve with dialysis. Pliskin *et al.*^21^ in a similar study comparing 16 well-dialysed patients with 12 controls did not find any significant difference. However, they noted a significant deficit in language abilities and intelligence in patients who scored well above the median on the BDI. In our study we also attempted to find out the effect of depression on the intellectual and neuropsychological functions by doing a correlation study of the BDI score with the performance IQ and the clinical subscales of the LNNB before and after renal transplant (Table 7). However, the BDI did not correlate with any of the subscales of the LNNB both pre- and post-transplant.

Analysis of results on the Life satisfaction scale (Table 8) showed that the majority in both the pre-transplant as well as the post-renal transplant groups scored in the high range. None of the patients scored low on the Life satisfaction scale. This is surprising especially as patients with ESRD undergo considerable stress due to the disease. Probably the support extended by the service in the form of attachment to the nearest unit in the station having a renal transplant facility, the availability of out-of-turn accommodation on medical grounds, continuation of employment and free hospitalization play a major role in achieving high scores on the Life satisfaction scale. Also, this may be a cultural variant where our patients seem generally satisfied with their lives notwithstanding the problems they face. We administered the Life satisfaction scale that seemed to answer the question about the quality of life satisfactorily. Quality of life has been linked to outcome in various studies. However, with our patients being generally satisfied with their lives, this finding could not be corroborated. The traditional philosophy in our country could also be the reason why the life satisfaction score is high. Most of the common coping methods that were seen have been reported earlier, i.e. patients accepted the situation because very little could be done, they told themselves that everything would work out fine and that the problem was not that important. Religious beliefs may also act as coping mechanisms and help in improving the quality of life in these patients.

## CONCLUSION

Neuropsychiatric evaluation of patients with ESRD on haemodialysis before and after transplantation revealed that 86.7% of the population was depressed before transplantation. Depression was significantly ameliorated in the post-transplant group. The BDI-II was found to be a sensitive and effective tool for detecting depression in this population. Significant differences were observed in the IQ performance as well as the language subscales of the LNNB before and after transplant. Life satisfaction scores also showed a significant improvement following renal transplant.
